# Early-Life Conditions and Cognitive Function in Middle-and Old-Aged Chinese Adults: A Longitudinal Study

**DOI:** 10.3390/ijerph17103451

**Published:** 2020-05-15

**Authors:** Lei Yang, Zhenbo Wang

**Affiliations:** 1 School of Ethnology and Sociology, Minzu University of China, Beijing 100081, China; lei.yang@muc.edu.cn; 2 Institute of Geographic Sciences and Natural Resources Research, Chinese Academy of Sciences, Beijing 100101, China

**Keywords:** childhood conditions, childhood socioeconomic status, cognitive function, early parental death, China Health and Retirement Longitudinal Study (CHARLS)

## Abstract

A range of previous studies have suggested that early-life conditions (ELCs) are associated with various health problems throughout life in Western societies. The aim of this study was to investigate whether, and how, early-life conditions predicted the level and trajectory of cognitive function in middle- and old-aged Chinese adults. Data were obtained from China Health and Retirement Longitudinal Study which comprised 16,258 adults at baseline. Cognitive function was assessed using mental intactness and episodic memory and ELCs were measured by early parental death, childhood socioeconomic status (SES), food deprivation, and childhood health. Growth curve modeling was used to examine the trajectory of cognitive function (three waves in a 6-year period)with particular attention paid to the effects of ELCs on cognition. The results show that early maternal death is associated with the baseline cognitive level among middle- and old-aged Chinese adults (β range between −0.44 and −0.35, *p* < 0.05), but that this association is also largely attenuated by adulthood education. Higher childhood SES predicts an enhanced level of baseline cognition in both age groups (β range between 0.08 and 1.27, *p* < 0.001), but only protects against cognitive decline at baseline in middle-aged adults. Participants who were less healthy during childhood tended to have lower cognitive performance than those who had enjoyed good health (β range between −0.36 and −0.14, *p* < 0.05). The results of this study highlight the detrimental impact of deleterious ELCs on cognitive function throughout later life.

## 1. Background

Health disparities are thought to originate in childhood. Accumulating evidence suggests that early-life conditions (ELCs), usually measured via childhood adversity, socioeconomic status (SES), and health status, are associated with a range of health problems in later life [1,2,3]. Previous work in this area has generally been founded within a life course epidemiological framework in order to demonstrate how ELCs affect later health. Research has shown that early parental death, childhood SES and health status can be associated with lower cognition, depression, and physical health problems in adulthood [4,5,6].

Cognition is one of the most important health variables and declining cognitive function with age is a major concern for middle- and old-aged adults globally [7]. Decreased cognitive function is associated with an increased risk of mortality, disability and poor quality-of-life [7]. It is well-documented that sociodemographic factors including sex [8], marital status [9], and residential area [10] as well as SES [11,12], physical health [13], chronic diseases [14], and health behaviors [15] predict cognition and its decline among old-aged adults.

Indeed, from a life course perspective, previous studies have suggested that ELCs are likely associated with cognition at middle and older ages. For instance, childhood SES is usually considered to be positively associated with cognitive function in middle- and old-age [12,16,17]. Childhood adversity has also been shown to be related to cognition (e.g., immediate memory, visuospatial construction, language, and delayed memory abilities) [18,19] and some research has even suggested that childhood abuse can be associated with better global cognition, memory, executive function, and processing speeds [20]. Childhood health is likely a robust indicator for orientation, word recall, and backward counting in later life [21,22,23]. Previous research has suggested that exposure to adverse ELCs was associated with increased anxiety levels and cognitive impairment as these variables disrupt the maturation of underlying brain networks [24,25]. The concept of cognitive reserve in this context postulates that individual differences in these processes, or in the neural networks underlying task performance, allow some individuals to cope better than others with brain damage [26].

It is clear that cognitive function will become less efficient with age, but both the level and the speed at which this decline occurs varies greatly among individuals [27]. It is the case that the impacts of traumatic events and socioeconomic disadvantages during childhood can accumulate throughout life on the basis of the cumulative disadvantage theory [28]. Although some previous studies have found an association between childhood SES and cognitive decline in later life in Western societies [11,29], it remains unclear whether the negative effects of ELCs on cognitive function persist from middle- to old-age in China.

In recent decades, China has experienced massive and historical socioeconomic changes, in particular throughout the 20th century (e.g., World War II and the Great Famine between 1959 and 1961). Certain cohorts of people might be more vulnerable to the effects of poor early-life experiences as they age. These rapid social changes will have exerted a number of influences on the early-life experiences of individuals. Indeed, compared with their counterparts in developed societies, many older Chinese adults experienced impoverished conditions, widespread social unrest, and upheaval during child- and adulthood [17]. Adverse life events, such as hunger and war (which might include parental death), could exert long-lasting impacts on the development of cognition throughout life. At the same time, dramatic socioeconomic changes in China after economic reforms in 1978 might have also mitigated or alleviated the effects of early-life conditions (ELCs) on health trajectories, but this requires further research. China therefore provides a unique case study for us to examine the possible long-term effects of early conditions on cognition throughout life. Although a few studies have investigated associations between ELCs and cognition in older Chinese adults, their main focus has either been on older people [30] or they have used limited measures to assess these conditions [31,32]. A limited amount of work has been afforded to understanding the trajectory of cognition due to ELCs, especially early parental death amongst middle- and old-aged Chinese adults. Thus, the added value of this study is that we focus on middle- and old- aged adults who have been subjected to more impoverished conditions than their counterparts in Western societies.

This research therefore aimed to study the associations between ELCs (i.e., early parental death, childhood SES, food deprivation, and childhood health) and the level and trajectory of cognitive function amongst middle- and old-aged Chinese adults on the basis of a large dataset of nationally representative longitudinal cohort data. This study also verified whether or not these associations differ by age group and persist into later life. We hypothesized that the effects of ELCs on cognition would be evident among middle- and old- aged adults in China.

## 2. Materials and Methods

### 2.1. Data and Measurements

The data used in this study were extracted from the China Health and Retirement Longitudinal Study (CHARLS), a nationally representative study of Chinese community-dwelling adults aged 45 years and older. The baseline survey used here was randomly selected from 150 counties using multistage probability sampling in 2011 and two follow-up surveys were conducted in 2013 and 2015. The initial life history survey reported here was performed in 2014 and collected detailed individual information (i.e., residence, demographic, family, education, health, work, and wealth) from childhood to adulthood. All data were collected using face-to-face computer-assisted personal interviews and had a response rate over 80% at baseline [33]. The survey was approved by the Ethical Review Committee of Peking University, and all participants signed written-informed consent forms. The life history survey was linked with the baseline sample and involved 23,701 participants. Participants who did not take part in the life event history survey in 2014 because of death or because they were lost to follow-up, were younger than 45 years of age, or because age or gender data were missing were dropped from the sample. The final analytical sample included 16,258 men and women aged between 45 and 101 years at baseline.

### 2.2. Cognitive Function

Consistent with the American Health and Retirement Survey (HRS), two composite measures of cognitive function were used in this study: episodic memory and mental intactness. The first of these, episodic memory, comprises immediate and delayed word recall. Word recall scores were based on the ability of a respondent to immediately repeat ten Chinese nouns that had been read to them in any order (i.e., immediate word recall, scored on a scale up to 10) as well as their ability to recall the same list of words four minutes later (i.e., delayed recall, scored on a scale up to 10). In line with previous studies [10,34], episodic memory scores were measured using average values for immediate and delayed recall. Similarly, mental intactness measures were based on mental status question components taken from the Telephone Interview of Cognitive Status (TICS) bank established to capture mental intactness. These questions assess participants’ ability to make seven serial subtractions from 100 (up to five times, scored on a scale up to five), naming the date (i.e., month, day, year, and season, scored on a scale up to four), the day of the week (scored either as zero or one), and redraw a picture that had just been seen (scored either as zero or one). Answers to these questions were then aggregated into a single mental intactness score that ranged up to 10; higher scores indicate better cognitive function.

### 2.3. ELCs

A set of four self-reported ELCs before 17 years of age were used in this study, early parental death, childhood SES (i.e., education and occupation of father), food deprivation, and childhood health. The first of these, early parental death, was assessed using early paternal death (yes or no) and early maternal death (yes or no). Childhood SES was measured by scoring educational attainment and occupation of the father in each case because males were always the core family supporter in the older Chinese society. Food deprivation was measured on the basis of food adequacy during childhood and self-rated health was divided into three categories, healthy, average, and less healthy.

### 2.4. Covariates

Scores and measurements for age, gender, living area (i.e., rural or urban), adulthood educational attainment and physical injury (yes or no) as well as marital status (married or not), Activity of Daily Living (ADL), number of chronic diseases (including high blood pressure, diabetes, cancer, lung disease, heart problem, stroke, psychiatric problem, arthritis, dyslipidemia, liver disease, kidney disease, digestive disease, asthma, and memory problems), and current health behaviors (smoking or drinking) were used as covariates. Measures for ADL in the CHARLS analysis comprised six basic physical activities, eating, dressing, bathing and showering, getting in and out of bed, using the toilet, and controlling urination and defecation. Thus, ADL was coded as zero if none of these six activities was impaired but was coded as one if one or more were impaired. All covariates were set as time-varying variable, except gender, adulthood educational attainment, and physical injury.

### 2.5. Statistical Methods

Multilevel growth curve modeling (MGCM) was used to estimate cognitive function age trajectories over time. Emphasis was placed on the effects of ELCs and so MGCM were designed to analyze the trajectory of repeated measures in longitudinal data. Two levels of panel data were used including the repeated measurement of cognitive function at Level 1 nested across individuals at Level 2. We used two-level hierarchical linear models to estimate age trajectories of cognitive function and encapsulated heterogeneity in these trajectories for ELCs.

The Level 1 model used here was defined as follows:

(1) $$y_{ti}=\beta_{0i}+\beta_{1i}Age_{ti}+e_{ti}.$$

The Level 2 model used here was defined including an intercept, as follows:

(2) $$\beta_{0i}=\gamma_{00}+\gamma_{01}ELC+\gamma_{02}Covariates+u_{0i}.$$

A model for the linear rate of change (age) was also included, as follows:

(3) $$\beta_{1i}=\gamma_{10}+\gamma_{11}ELC+u_{1i}.$$

Full information maximum likelihood estimation was employed to effectively handle missing values [11]. Thus, the ‘time’ variable used in this study was chronological age because change in cognition is known to be closely related to age [11]. Age was centered at the mean for each group at baseline in order to make the model stable [35].

As in previous works [17,32], we included two age groups (aged between 45 years of age and 59 years of age and aged over 60) in order to separately calculate the difference between middle- and old-aged adults. Thus, four models stratified on the basis of these age groups were generated step-by-step to investigate whether or not the interplay between ELCs and cognitive function was mediated by adulthood SES and other covariates. Age and gender only were included in Model 1, while adulthood educational attainment was adjusted into Model 2. Additional covariates were included in Model 3, while interaction terms between ELCs and age measuring the age trajectory of cognition were added to Model 4. We do not report results stratified by gender as no significant interaction effects were recovered (i.e., 0.07 < *p* < 0.89) with respect to ELCs in this case. Sensitivity analysis including the participants due to death or lost to follow-up in 2014 produced compared conclusions. Correlations of age and mean scores of mental intactness and episodic memory in 2011 were available in Supplementary Figures S1 and S2. All analyses were conducted using the statistical software Stata 16.0 (Stata Corp., College Station, TX, USA).

## 3. Results

Descriptive sample statistics at baseline are presented in Table 1. These data show that, across the whole sample, mean scores for mental intactness and episodic memory were 6.4 and 3.5, respectively. The results show that middle-aged adults tend to have significantly higher cognitive scores than their older counterparts. Data also show that between 10% and 15% of respondents had experienced either paternal or maternal death before 17 years of age; adults aged between 45 years of age and 59 years of age had experienced a lower proportion of early parental death than older adults. Approximately 46% of middle-aged adults’ fathers were able to read or write, while the percentage of old-aged adults with a literate father was 34%. Almost 30% of respondents were food deprived during their childhood, while around 13% of Chinese adults self-reported being less healthy during early-life. Mean values for middle- and old-aged adults were 52 years of age and 68 years of age, respectively.

Table 2 presents the mean scores of cognitive function in different waves by early-life conditions. The mental intactness and episodic memory declined with early-life conditions from 2011 to 2015 and participants with disadvantageous early-life conditions had a lower cognitive function compared with their counterparts. For instance, the mean scores of mental intactness of adults who experienced paternal death or not was 6.6 or 6.2 in 2011, respectively. Adults with an illiterate father had lower mental intactness (6.0) than those whose father was able to read or write (7.1). Older adults whose paternal occupation was non-agricultural scored lower in cognitive function than those whose paternal occupation was agricultural (e.g., 4.2 vs. 3.4 in wave 1). Similar patterns were also found among other early-life conditions.

Estimates for the effect of ELCs on cognitive function across the two age groups are presented in Table 3 and Table 4, respectively. In terms of mental intactness (Table 3, Model 1), adults who had experienced early maternal death scored lower (β = −0.44 for younger adults or −0.35 for older adults, *p* < 0.001) compared with those who had not experienced this trauma. The results reveal that early paternal death was generally not associated with baseline mental intactness at baseline in either middle- or old-aged adults. Participants with a literate father, whose paternal occupation was non-agricultural and who had experienced food adequacy (i.e., middle-aged adults) scored between 0.28 (*p* < 0.001) and 1.27 (*p* < 0.001) higher for baseline cognition than their counterparts. Similarly, participants who were less healthy during childhood attained lower baseline mental intactness scores, between −0.36 (*p* < 0.001) and −0.19 (*p* < 0.01), than for their health status. Adulthood education was included in Model 2; these results show that early maternal death and food adequacy were not statistically significant while paternal education and employment as well as self-rated health remained significant. A higher level of adulthood education was linked to better cognition at baseline in this model; indeed, results from Model 3 were comparable with those recovered from Model 2. For cognitive function change, Model 4 presents coefficients used to assess whether or not ELCs can be associated with temporal changes in cognitive function. These data show that, for both age groups, interactions between age and ELC indicators were non-significant apart from food adequacy; this result indicates that an individual experienced slower cognitive change over time if they were adequate in food during childhood.

Results for episodic memory (Table 4, Model 1) show that early paternal death was not related to the baseline cognition intercept in adults aged between 45 years of age and 59 years of age, but was associated with lower word recall scores (0.20, *p* < 0.001) among adults aged more than 60 years of age. Data show that early maternal death predicted lower episodic memory at baseline, while enhanced paternal education, a non-agricultural job, food adequacy, and good childhood health status were generally associated with better episodic memory in both age groups. The results also show that early maternal death predicted 0.20 (*p* < 0.01) or 0.20 (*p* < 0.001) lower baseline episodic memory scores than those seen in individuals who had not experienced this trauma across the two age groups. Indeed, increased childhood SES (i.e., non-agricultural paternal employment and a literate education) values were associated with higher baseline memory scores, between 0.23 (*p* < 0.001) and 0.65 (*p* < 0.001). Data recovered from Model 2 and Model 3 (Table 4) reveal that early maternal death and food adequacy (in the younger age group) were not associated with baseline word recall scores while other ELCs encompassing paternal education and employment as well as childhood self-rated health remained statistically significant. For cognitive function change, results from Model 4 show that paternal non-agricultural employment was associated with a slower rate of change in word recall of 0.02 in middle-aged adults over time. Interactions between age and all indicators of ELCs were not statistically significant in old-aged individuals; this indicates that ELCs are not related to cognitive change over time.

## 4. Discussion

Longitudinal survey data collected between 2011 and 2015 from a large cohort study of middle- and old-aged Chinese adults were used to examine whether or not ELCs are associated with both baseline level and changes in cognitive function over time. We also investigated whether adulthood factors attenuated this association. The results suggest that early parental death can generally be associated with a lower baseline cognition level, but that this effect is largely attenuated by education in adulthood. Higher paternal education, non-agricultural job, and childhood good health are also all robust predictors for baseline cognition. Indeed, both a non-agricultural paternal job and food adequacy in childhood can also be associated with a slower rate of cognitive change during more than four years of follow-up with a sample of only middle-aged adults. The results of this analysis suggest that early-life circumstances during childhood are critical to long-term health development. In other words, early-life risk factors shape both short- and long-term health trajectories and outcomes [28]. This study contributes to the current life-course literature by demonstrating the long-lasting effects of ELCs on both baseline level and rate of change in cognitive function amongst middle- and old-aged adults in a middle-income country such as China.

The unadjusted model results presented here for the relationship between childhood parental death and cognition are in line with a few other notable studies based on Western societies where early parental death has been associated with lower adult cognitive function [36,37]. Evidence suggest that adversity earlier in life (i.e., parental death during childhood) may lead to higher subsequent vulnerability to particular forms of psychiatric disorders because of rapid brain development at these ages and risks that persist into later life [38,39]. The results presented in this study suggest, however, that such effects disappear when adulthood education and other factors are adjusted; results suggest that adulthood factors, especially educational attainment, play a more important role in the development of cognitive function amongst middle- and old-aged Chinese adults. This finding is consistent with growing numbers of studies which have also shown that education in adulthood can explain a substantial proportion of the link between ELCs and later life cognition [16,23]. Nevertheless, the results do contradict one study carried out in the United States that suggested early parental death remains a significant factor in later life even after adjusting for adulthood SES and other variables [37]. This discrepancy might be due to a different conceptualization of childhood conditions and cognitive assessment, analytical methods, or cultural context [40]. It is also possible that children who experience early parental death are taken care of by other family members, a common phenomenon decades ago in China when multiple generations tended to live together [41]. This might have served as a buffering resource for health outcomes amongst Chinese adults who lost their parent(s) during childhood [42].

Childhood SES (usually measured using parental SES) has also been shown to be a robust predictor of health in middle and old age. Indeed, consistent with our results, a growing body of previous research in several Western countries supports the presence of positive links between childhood SES and middle- and old-age cognitive function [6,40,43]. This is also the case in China [17,32]. We show that both paternal education and employment can be robustly associated with better cognitive function in both middle- and old-aged individuals, although adulthood education as well as sociodemographic and health factors were adjusted. It has also been suggested that childhood SES contributes to cognitive progress in early life via the quality and quantity of parent–child interactions and verbalizations [44]. Thus, poor childhood SES may directly influence cognition in later life via brain maturation in childhood and adolescence, resulting in less efficient function [11]. Previous research has also suggested that childhood SES indirectly influences cognition in adults because this variable also impacts adulthood SES. This means that adverse childhood circumstances often have deleterious impacts on adult educational achievement and employment [17].

The results of this analysis show that models produce mixed results when assessing food deprivation (food adequacy) across different age groups versus measures of cognition. Unadjusted model data show that adequate food in childhood can predict better cognitive performance in middle-aged adults, even though this association was not significant in all models for the old-aged. In terms of episodic memory, food adequacy was generally not associated with cognitive performances amongst adults aged between 45 and 59, while it was related to lower word recall scores among older individuals (60+ years of age). This anomalous finding might be due to survival effects; people who have survived severe food deprivation tend to be healthier than their counterparts. In line with previous studies [6,8], poorer childhood self-rated health was generally associated, in this analysis, with lower cognition levels amongst middle- and old-aged adults. It is clear that children with poorer health earlier in life will go onto experience significantly lower educational attainment, poorer health, and lower SES as adults [45]. Childhood health may thus influence initial adult health and SES before then contributing to later-life conditions.

More importantly, the results of this study suggest that parental occupation and food adequacy are both associated with a slower rate of cognitive decline in the younger age group, which implies diverging cognitive trajectories depending on agricultural versus non-agricultural paternal employment as well as food adequacy and deprivation in groups aged between 45 years of age and 60 years of age. We did not find any association between ELCs and cognitive change over time in the old-aged group. The findings of this study therefore suggest that more advantageous childhood SES protects against cognitive decline before 60 years of age but appears not to provide a reserve, or buffer, against decline after this age, at least in China. These ELC trajectories of cognition neither diverge nor converge with age after aged 60 years. Our results corroborate earlier studies which have found no association between childhood SES and cognitive function rate-of-decline amongst older adults [11,12,46,47,48]. Indeed, the older cohort (over 60 years of age) in our analyses were mostly born between the decade commencing in 1910 and the decade commencing in 1950 and so experienced a range of important historical events (i.e., The Second World War between 1937 and 1945, the civil war between 1945 and 1949, and the Great Famine between 1959 and 1961) in China. Numerous individuals will also have spent their childhoods experiencing malnutrition and turbulence before then facing rapid postwar socioeconomic transitions after the founding of the People’s Republic of China in 1949. This context may have acted to weaken the relationships between childhood SES and health trajectories. Substantial improvements in living standards within China over the past few decades may have also acted to alleviate the effects of childhood SES on later health amongst older individuals. This scenario implies that the link between childhood SES and the health outcomes of older adults might, in fact, be weaker for Chinese individuals who have experienced improvements in their living standards in adulthood.

The strength of this study is that it is based on a longitudinal dataset and therefore encompasses a large sample size. This enabled ELCs to be assessed using a range of indicators including early parental death, childhood SES and health as well as food deprivation. Our use of growth curve modeling has enabled us to investigate cognitive trajectories due to ELCs. At the same time, it is also important to note the limitations of this study; in the first place, markers of ELCs were collected by self-reporting which might have introduced recall bias. This may have resulted in an underestimation of the association between ELCs and cognition. We were also not able to include some protective (e.g., resilience, ability to cope with stress, adaptation) and genetic factors related to cognition in this study because this information was not present in the dataset. Further studies are warranted in the future.

## 5. Conclusions

The results of this analysis reveal that even though early maternal death is associated with baseline cognitive level in middle- and old-aged Chinese adults, this association is largely mitigated by adulthood education. Childhood SES predicts a higher baseline cognitive level in both age groups, but only protects against decline in middle-aged adults. Participants who were less healthy during childhood also tended to exhibit a lower cognitive performance later in life. Policy attention should therefore be afforded to disadvantaged children across China in order to mitigate possible long-term detrimental health impacts in later decades.

## Figures and Tables

**Table 1:** Baseline sample descriptive statistics (N = 16,258).

Variable	Total Mean/%	Aged 45–59 Years Mean/%	Aged 60 + Mean/%	*p* Value for Age Group Difference
Mental intactness	6.4 (2.9)	6.8 (2.7)	5.9 (3.1)	<0.001
Episodic memory	3.5 (1.7)	3.8 (1.7)	3.1 (1.7)	<0.001
Early paternal death				<0.001
No	84.9	88.9	78.7	
Yes	15.1	11.1	21.3	
Early maternal death				<0.001
No	90.1	93.1	85.7	
Yes	9.9	6.9	14.3	
Paternal education				<0.001
Illiterate	59.1	54.3	65.9	
Able to read or write	40.9	45.7	34.1	
Paternal employment				<0.001
Agricultural	80.4	78.5	83.2	
Non-agricultural	19.6	21.5	16.8	
Food adequacy				<0.001
Yes	29.3	32.1	25.4	
No	70.7	67.9	74.6	
Childhood self-rated health				0.06
Healthy	35.2	35.6	34.6	
Average	51.6	50.9	52.7	
Less healthy	13.2	13.5	12.7	
Age	59 (9.5)	52 (4.5)	68 (6.5)	/
Gender				/
Male	48.6	47.7	49.9	
Female	51.4	52.3	50.1	
Adulthood education	3.3 (1.9)	3.7 (1.9)	2.8 (1.8)	<0.001
Marital status				<0.001
Married	87.7	94.4	79.2	
Not married	12.3	5.6	20.8	
Living areas				0.30
Rural	63.4	63.0	63.9	
Urban	36.6	37.0	36.1	
ADL limitation				<0.001
Yes	16.3	10.7	23.5	
No	83.7	89.3	76.5	
Adulthood physical injury				0.45
Yes	8.5	8.4	8.7	
No	91.5	91.6	91.3	
Number of chronic diseases	1.5 (1.4)	1.3 (1.3)	1.7 (1.5)	<0.001
Current smoker				<0.001
No	60.1	61.8	58.0	
Yes	39.9	38.2	42.0	
Current drinker				<0.001
No	66.6	64.4	69.4	
Yes	33.4	35.6	30.6	

**Table 2:** Mean scores of cognitive function (2011–2015) by early-life conditions.

Variable	Mental Intactness			Episodic Memory		
Year	Wave 1	Wave 2	Wave 3	Wave 1	Wave 2	Wave 3
Early paternal death (*p* < 0.000) ^a^						
No	6.6	6.5	6.3	3.6	3.5	3.3
Yes	6.2	6.1	5.8	3.3	3.1	2.8
Early maternal death (*p* < 0.000) ^b^						
No	6.5	6.4	6.3	3.6	3.5	3.2
Yes	5.9	5.8	5.5	3.2	3.1	2.8
Father’s education (*p* < 0.000) ^c^						
Illiterate	6.0	5.9	5.6	3.4	3.2	2.9
Can read or writing	7.1	7.0	6.7	3.9	3.8	3.5
Father’s job (*p* < 0.000) ^d^						
Agricultural	6.2	6.1	5.8	3.4	3.3	3.0
Non-agricultural	7.6	7.4	7.1	4.2	4.1	3.9
Food adequacy (*p* < 0.000) ^e^						
Yes	6.6	6.5	6.3	3.7	3.5	3.3
No	6.3	6.2	5.9	3.5	3.4	3.1
Childhood self-rated health (*p* < 0.000) ^f^						
Healthy	6.6	6.5	6.3	3.7	3.5	3.3
Average	6.4	6.3	6.0	3.5	3.3	3.2
Less healthy	6.0	5.9	5.7	3.4	3.2	3.0

a,b,c,d,e,f: *p* < 0.000 indicates significant differences by each early-life conditions in each wave.

**Table 3:** Associations between early-life conditions (ELC) and mental intactness scores for middle- and old-aged Chinese adults.

Variables	Aged 45–59 Years				Aged 60 Years+			
	β, Model 1	β, Model 2	β, Model 3	β, Model 4	β, Model 1	β, Model 2	β, Model 3	β, Model 4
Early paternal death (no)								
Yes	−0.10	0.09	0.10	0.10	−0.10	0.14	0.18	0.07
Early maternal death (no)								
Yes	−0.44 ***	−0.15	−0.13	−0.19 *	−0.35 **	−0.09	−0.09	−0.03
Paternal education (illiterate)								
Able to read and write	0.54 ***	0.26 ***	0.23 ***	0.21 ***	0.80 ***	0.36 ***	0.33 ***	0.27 **
Paternal employment (agricultural)								
Non-agricultural	0.92 ***	0.28 ***	0.15 *	0.11 *	1.27 ***	0.47 ***	0.29 ***	0.13 **
Food adequacy (no)								
Yes	0.28 ***	0.05	0.02	0.05	0.01	−0.09	−0.11	−0.34
Childhood self-rated health (healthy)								
Average	−0.08	−0.01	−0.01	0.01	−0.19 *	−0.14 *	−0.11	−0.11
Less healthy	−0.36 ***	−0.26 ***	−0.19 *	−0.19 *	−0.36 **	−0.27 *	−0.22 *	−0.22 ***
Early paternal death * age				−0.01				0.01
Early maternal death * age				−0.03				−0.01
Paternal education * age				0.01				0.01
Paternal employment * age				0.02				0.01
Food adequacy * age				0.03 **				0.01
Age	−0.05 ***	−0.02 **	−0.01 **	−0.02	−0.10 ***	−0.08 ***	−0.07 ***	−0.08 ***
Female (male)	−1.31 ***	−0.52 ***	−0.58 ***	−0.58 ***	−1.86 ***	−0.90 ***	−0.91 ***	−0.91 ***
Education		0.64 ***	0.61 ***	0.61 ***		0.76 ***	0.71 ***	0.71 ***
Marital status (married)								
Not married			−0.31 **	−0.30 **			−0.24 **	−0.24 **
Rural (urban)			−0.47 ***	−0.47 ***			−0.60 ***	−0.60 ***
ADL			−0.31 ***	−0.30 ***			−0.45 ***	−0.45 ***
Adulthood physical limitation (no)			−0.27 ***	−0.27 **			0.03	0.03
Number of chronic diseases			−0.03 *	−0.03 *			0.01	0.01
Current smoker (no)			−0.09	−0.09			−0.01	−0.01
Current drinker (no)			0.08	0.08 *			0.01	0.01
-Log likelihood	44,140.83	43,085.56	42,290.61	42,282.05	28,944.13	28,147.55	27,841.16	27,839.66

β: Coefficient; Reference groups in parentheses (* *p* < 0.05; ** *p* < 0.01; *** *p* < 0.001); Model 1: ELCs + age + gender; Model 2: ELCs + age + gender + education; Model 3: ELCs + age + gender + education + other covariates; Model 4: ELCs + age + gender + education + other covariates + interactions between ELCs and age.

**Table 4:** Associations between ELCs and episodic memory scores for middle- and old-aged Chinese adults.

Variables	Aged 45–59 Years				Aged 60 Years+			
	β, Model 1	β, Model 2	β, Model 3	β, Model 4	β, Model 1	β, Model 2	β, Model 3	β, Model 4
Early paternal death (no)								
Yes	−0.03	0.06	0.07	0.16	−0.20 ***	−0.10 *	−0.09 *	−0.10 *
Early maternal death (no)								
Yes	−0.20 **	−0.06	−0.06	−0.08	−0.20 ***	−0.10	−0.10	−0.09
Paternal education (illiterate)								
Able to read and write	0.23 ***	0.09 **	0.08 *	0.03 *	0.33 ***	0.16 ***	0.15 ***	0.15 ***
Paternal employment (agricultural)								
Non-agricultural	0.65 ***	0.35 ***	0.31 ***	0.56 ***	0.65 ***	0.34 ***	0.27 ***	0.27 ***
Food adequacy (no)								
Yes	0.16 ***	0.04	0.03	0.01	−0.05	−0.09 *	−0.10 *	−0.10 *
Childhood self-rated health (healthy)								
Average	−0.12 ***	−0.08 *	−0.07 *	−0.07 *	−0.17 ***	−0.15 ***	−0.14 **	−0.14 ***
Less healthy	−0.21 ***	−0.17 **	−0.14 *	−0.14 *	−0.15 *	−0.11	−0.07	−0.07
Early paternal death * age				0.01				0.01
Early maternal death * age				0.01				−0.01
Paternal education * age				−0.01				−0.01
Paternal employment * age				0.02 **				−0.01
Food adequacy * age				−0.01				−0.10
Age	−0.05 ***	−0.03 ***	−0.03 ***	−0.05 **	−0.08 ***	−0.07 ***	−0.07 ***	−0.07 ***
Female (male)	−0.14 ***	0.24 ***	0.28 ***	0.28 ***	−0.25 ***	0.13 **	0.08	0.08
Education		0.31 ***	0.30 ***	0.30 ***		0.30 ***	0.28 ***	0.28 ***
Marital status (married)								
Not married			−0.13 *	−0.13 *			−0.10 *	−0.10 *
Rural (urban)			−0.14 ***	−0.14 ***			−0.24 ***	−0.24 ***
ADL			−0.15 ***	−0.14 ***			−0.16 ***	−0.16 ***
Adulthood physical limitation (no)			−0.04	−0.04			−0.03	−0.03
Number of chronic diseases			−0.01	−0.01			−0.03 *	−0.02 *
Current smoker (no)			0.01	0.01			−0.10 *	−0.10 *
Current drinker (no)			0.10 ***	0.10 **			0.07	0.07
-Log likelihood	35,945.26	35,327.65	34,661.79	34,657.78	22,926.31	22,544.16	22,328.90	22,327.79

β: Coefficient; Reference groups in parentheses (* *p* < 0.05; ** *p* < 0.01; *** *p* < 0.001); Model 1: ELCs + age + gender; Model 2: ELCs + age + gender + education; Model 3: ELCs + age + gender + education + other covariates; Model 4: ELCs + age + gender + education + other covariates + interactions between ELCs and age.

## Supplementary Materials

The following are available online at https://www.mdpi.com/1660-4601/17/10/3451/s1, Figure S1: Correlation of age and mental intactness (mean scores) in 2011, Figure S2: Correlation of age and episodic memory (mean scores) in 2011.

## References

[1] Hailey Maier E., Lachman M.E. (2000). Consequences of early parental loss and separation for health and well-being in midlife. Int. J. Behav. Dev..

[2] Pavela G., Latham K. (2016). Childhood conditions and multimorbidity among older adults. J. Gerontol. Ser. B Psychol. Sci. Soc. Sci..

[3] McEniry M. (2013). Early-life conditions and older adult health in low- and middle-income countries: A review. J. Dev. Orig. Health Dis..

[4] Laditka J.N., Laditka S.B. (2018). Adverse Childhood Circumstances and Functional Status Throughout Adult Life. J. Aging Health.

[5] St Clair M.C., Croudace T., Dunn V.J., Jones P.B., Herbert J., Goodyer I.M. (2015). Childhood adversity subtypes and depressive symptoms in early and late adolescence. Dev. Psychopathol..

[6] Luo Y., Waite L.J. (2005). The impact of childhood and adult SES on physical, mental, and cognitive well-being in later life. J gerontol. Ser. B psychol. Sci. Soc. Sci..

[7] Zaninotto P., Batty G.D., Allerhand M., Deary I.J. (2018). Cognitive function trajectories and their determinants in older people: 8 years of follow-up in the English longitudinal study of ageing. J. Epidemiol. Community Health.

[8] Greenfield E.A., Moorman S.M. (2019). Childhood socioeconomic status and later life cognition: Evidence from the wisconsin longitudinal study. J. Aging Health.

[9] Liu H., Zhang Y., Burgard S.A., Needham B.L. (2019). marital status and cognitive impairment in the united states: Evidence from the national health and aging trends study. Ann. Epidemiol..

[10] Lei X., Smith J.P., Sun X., Zhao Y. (2014). Gender differences in cognition in china and reasons for change over time: Evidence from charls. J. Econ. Ageing.

[11] Lyu J., Burr J.A. (2016). Socioeconomic Status Across the Life Course and Cognitive Function Among Older Adults:An Examination of the Latency, Pathways, and Accumulation Hypotheses. J. Aging Health.

[12] Staff R.T., Chapko D., Hogan M.J., Whalley L.J. (2016). Life course socioeconomic status and the decline in information processing speed in late life. Soc. Sci. Med..

[13] Clouston S.A., Brewster P., Kuh D., Richards M., Cooper R., Hardy R., Rubin M.S., Hofer S.M. (2013). The dynamic relationship between physical function and cognition in longitudinal aging cohorts. Epidemiol. Rev..

[14] Kumar H., Arokiasamy P., Selvamani Y. (2019). Socioeconomic disadvantage, chronic diseases and their association with cognitive functioning of adults in india: A multilevel analysis. J. Popul. Ageing.

[15] North T.-L., Palmer T.M., Lewis S.J., Cooper R., Power C., Pattie A., Starr J.M., Deary I.J., Martin R.M., Aihie Sayer A. (2015). Effect of smoking on physical and cognitive capability in later life: A multicohort study using observational and genetic approaches. BMJ Open.

[16] Kaplan G.A., Turrell G., Lynch J.W., Everson S.A., Helkala E.-L., Salonen J.T. (2001). Childhood socioeconomic position and cognitive function in adulthood. Int. J. Epidemiol..

[17] Zhang Z., Liu J., Li L., Xu H. (2018). The Long Arm of Childhood in China: Early-Life Conditions and Cognitive Function Among Middle-Aged and Older Adults. J. Aging Health.

[18] Petkus A.J., Lenze E.J., Butters M.A., Twamley E.W., Wetherell J.L. (2018). Childhood Trauma Is Associated With Poorer Cognitive Performance in Older Adults. J. Clin. Psychiatr..

[19] Schuitevoerder S., Rosen J.W., Twamley E.W., Ayers C.R., Sones H., Lohr J.B., Goetter E.M., Fonzo G.A., Holloway K.J., Thorp S.R. (2013). A meta-analysis of cognitive functioning in older adults with PTSD. J. Anxiety Disord..

[20] Feeney J., Kamiya Y., Robertson I.H., Kenny R.A. (2013). Cognitive function is preserved in older adults with a reported history of childhood sexual abuse. J. Trauma. Stress.

[21] Wang Q., Zhang H., Rizzo J.A., Fang H. (2018). The Effect of Childhood Health Status on Adult Health in China. Int. J. Environ. Res. Public Health.

[22] Kobayashi L.C., Glymour M.M., Kahn K., Payne C.F., Wagner R.G., Montana L., Mateen F.J., Tollman S.M., Berkman L.F. (2017). Childhood deprivation and later-life cognitive function in a population-based study of older rural South Africans. Soc. Sci. Med..

[23] Case A., Paxson C. (2008). Height, health, and cognitive function at older ages. Am. Econ. Rev..

[24] Maccari S., Krugers H.J., Morley-Fletcher S., Szyf M., Brunton P.J. (2014). The consequences of early-life adversity: Neurobiological, behavioural and epigenetic adaptations. J. Neuroendocrinol..

[25] Chen Y., Baram T.Z. (2016). Toward Understanding How Early-Life Stress Reprograms Cognitive and Emotional Brain Networks. Neuropsychopharmacol. Off. Publ. Am. Coll. Neuropsychopharmacol..

[26] Stern Y. (2009). Cognitive reserve. Neuropsychologia.

[27] Hartshorne J.K., Germine L.T. (2015). When does cognitive functioning peak? The asynchronous rise and fall of different cognitive abilities across the life span. Psychol. Sci..

[28] Dannefer D. (2003). Cumulative advantage/disadvantage and the life course: Cross-fertilizing age and social science theory. J. Gerontol. Ser. B.

[29] Barnes L.L., Wilson R.S., Everson-Rose S.A., Hayward M.D., Evans D.A., de Mendes Leon C.F. (2012). Effects of early-life adversity on cognitive decline in older African Americans and whites. Neurology.

[30] Wen M., Gu D. (2011). The effects of childhood, adult, and community socioeconomic conditions on health and mortality among older adults in China. Demography.

[31] Zhang Z., Gu D., Hayward M.D. (2010). Childhood nutritional deprivation and cognitive impairment among older Chinese people. Soc. Sci. Med..

[32] Sha T., Yan Y., Cheng W. (2018). Associations of childhood socioeconomic status with mid-life and late-life cognition in Chinese middle-aged and older population based on a 5-year period cohort study. Int. J. Geriatr. Psychiatr..

[33] Zhao Y., Hu Y., Smith J.P., Strauss J., Yang G. (2012). Cohort profile: The china health and retirement longitudinal study (CHARLS). Int. J. Epidemiol..

[34] McArdle J.J., Fisher G.G., Kadlec K.M. (2007). Latent variable analyses of age trends of cognition in the health and retirement study, 1992–2004. Psychol. Aging.

[35] Feinian C., Yang Y., Guangya L. (2010). Social Change and Socioeconomic Disparities in Health over the Life Course in China: A Cohort Analysis. Am. Sociol. Rev..

[36] Ritchie K., Jaussent I., Stewart R., Dupuy A.-M., Courtet P., Malafosse A., Ancelin M.-L. (2011). Adverse childhood environment and late-life cognitive functioning. Int. J. Geriatr. Psychiatr..

[37] Norton M.C., Østbye T., Smith K.R., Munger R.G., Tschanz J.T. (2009). Early parental death and late-life dementia risk: Findings from the Cache County Study. Age Ageing.

[38] Rudolph K.D., Flynn M. (2007). Childhood adversity and youth depression: Influence of gender and pubertal status. Dev. Psychopathol..

[39] Yehuda R., Golier J.A., Halligan S.L., Harvey P.D. (2004). Learning and memory in Holocaust survivors with posttraumatic stress disorder. Biol. Psychiatr..

[40] Aartsen M.J., Cheval B., Sieber S., Van der Linden B.W., Gabriel R., Courvoisier D.S., Guessous I., Burton-Jeangros C., Blane D., Ihle A. (2019). Advantaged socioeconomic conditions in childhood are associated with higher cognitive functioning but stronger cognitive decline in older age. Proc. Natl. Acad. Sci. USA.

[41] Yang F.A.N., Lou V.W.Q. (2016). Childhood adversities, urbanisation and depressive symptoms among middle-aged and older adults: Evidence from a national survey in China. Ageing Soc..

[42] Yang L., Hu Y., Silventoinen K., Martikainen P. (2019). Childhood adversity and depressive symptoms among middle-aged and older Chinese: Results from China health and retirement longitudinal study. Aging Ment. Health.

[43] Fors S., Lennartsson C., Lundberg O. (2009). Childhood living conditions, socioeconomic position in adulthood, and cognition in later life: Exploring the associations. J. Gerontol. Ser. B.

[44] Zeki Al Hazzouri A., Haan M.N., Galea S., Aiello A.E. (2011). Life-course exposure to early socioeconomic environment, education in relation to late-life cognitive function among older mexicans and mexican americans. J. Aging Health.

[45] Case A., Fertig A., Paxson C. (2005). The lasting impact of childhood health and circumstance. J. Health Econ..

[46] Brewster P.W.H., Melrose R.J., Marquine M.J., Johnson J.K., Napoles A., MacKay-Brandt A., Farias S., Reed B., Mungas D. (2014). Life experience and demographic influences on cognitive function in older adults. Neuropsychology.

[47] Everson-Rose S.A., de Mendes Leon C.F., Bienias J.L., Wilson R.S., Evans D.A. (2003). Early Life Conditions and Cognitive Functioning in Later Life. Am. J. Epidemiol..

[48] Ericsson M., Lundholm C., Fors S., Dahl Aslan A.K., Zavala C., Reynolds C.A., Pedersen N.L. (2017). Childhood social class and cognitive aging in the Swedish Adoption/Twin Study of Aging. Proc. Natl. Acad. Sci. USA.

