# Supplementary material for: Early-Life Conditions and Cognitive Function in Middle-and Old-Aged Chinese Adults: A Longitudinal Study
Source: Int J Environ Res Public Health. 2020 May 15;17(10):3451. doi: 10.3390/ijerph17103451 (PMC7277849; doi:10.3390/ijerph17103451)
Supplement: Supplementary file 1 [file ijerph-17-03451-s001.pdf]

Early-Life Conditions and Cognitive Function in Middle- and Old-Aged Chinese Adults: A Longitudinal Study

Lei Yang [1] and Zhenbo Wang [2,*]

[1] School of Ethnology and Sociology, Minzu University of China, Beijing 100081, China; lei.yang@muc.edu.cn

[2] Institute of Geographic Sciences and Natural Resources Research, Chinese Academy of Sciences, Beijing 100101, China

* Correspondence: wangzb@igsnrr.ac.cn

Received: 28 March 2020; Accepted: 13 May 2020; Published: date

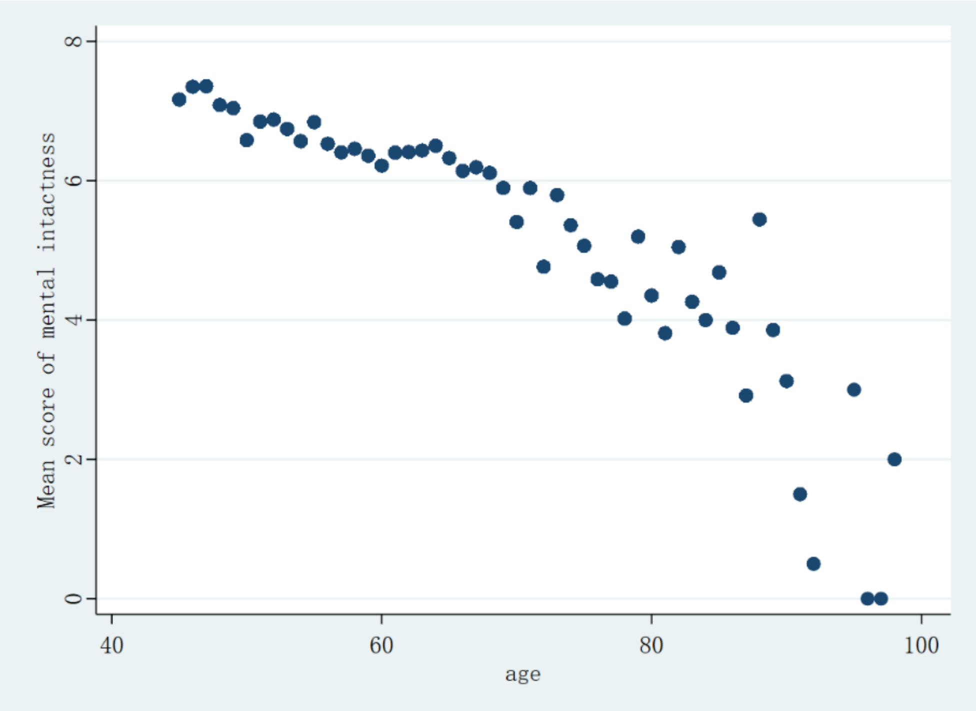

Figure S1. Correlation of age and mental intactness (mean scores) in 2011.

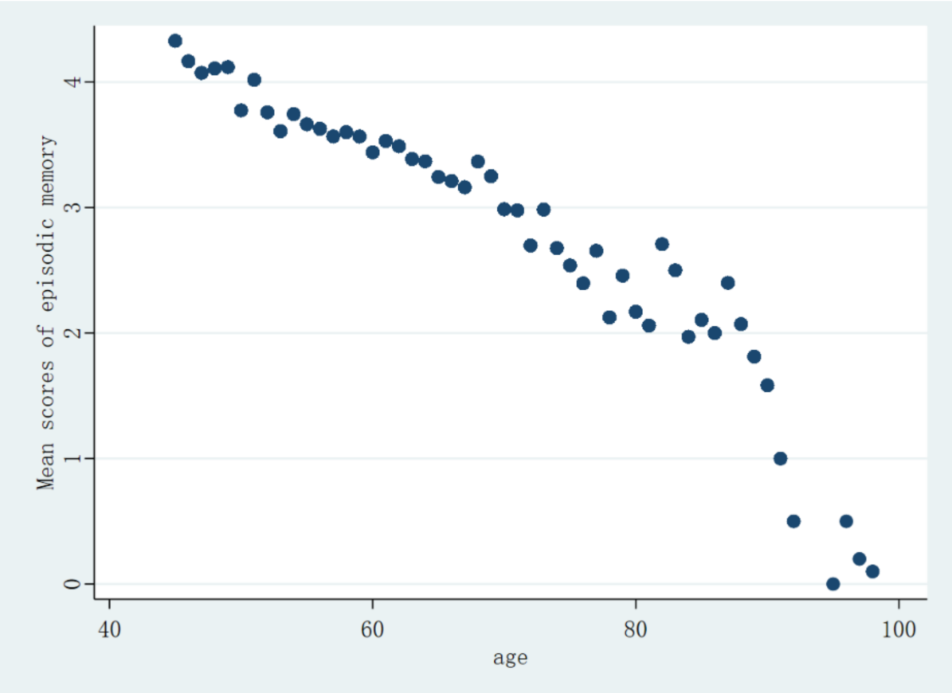

Figure S2. Correlation of age and episodic memory (mean scores) in 2011.
